# Low‐density lipoprotein cholesterol lowering in real‐world patients treated with evolocumab

**DOI:** 10.1002/clc.23600

**Published:** 2021-03-24

**Authors:** Nihar R. Desai, Rolin L. Wade, Pin Xiang, Lionel Pinto, Sasikiran Nunna, Xin Wang, Jason Exter, Katherine E. Mues, Mohdhar Habib, Chi‐Chang Chen

**Affiliations:** ^1^ Yale School of Medicine New Haven Connecticut USA; ^2^ IQVIA Plymouth Meeting Pennsylvania USA; ^3^ Amgen Inc. Thousand Oaks California USA; ^4^ Boehringer Ingelheim Pharmaceuticals, Inc. Ridgefield Connecticut USA; ^5^ Bristol Myers Squibb, Inc. Lawrenceville New Jersey USA

**Keywords:** atherosclerotic cardiovascular disease, LDL‐C, real‐world evidence

## Abstract

**Background:**

Low‐density lipoprotein cholesterol (LDL‐C) is a risk factor for atherosclerotic cardiovascular disease (ASCVD). There are limited real‐world data on LDL‐C lowering with evolocumab in United States clinical practice.

**Hypothesis:**

We assessed LDL‐C lowering during 1 year of evolocumab therapy.

**Methods:**

This retrospective cohort study used linked laboratory (Prognos) and medical claims (IQVIA Dx/LRx and PharMetrics Plus^®^) data. Patients with a first fill for evolocumab between 7/1/2015 and 10/31/2019 (index event) and LDL‐C ≥ 70 mg/dL were included (overall cohort; N = 5897). Additionally, a patient subgroup with a recent myocardial infarction (MI) within 12 months (median 130 days) before the first evolocumab fill was identified (N = 152). Reduction from baseline LDL‐C was calculated based on the lowest LDL‐C value recorded during a 12‐month follow‐up period.

**Results:**

The mean (SD) age was 65 (10) years; 61.9% of patients had ASCVD diagnoses and 70.7% of patients were in receipt of lipid‐lowering therapy. Following evolocumab treatment, changes in LDL‐C from baseline were −60% in the overall cohort (median [interquartile range (IQR)] 146 [115–180] mg/dL to 58 [36–84] mg/dL) and −65% in the recent MI subgroup (median [IQR] 137 [109–165] mg/dL to 48 [30–78] mg/dL). In the overall cohort and recent MI subgroup, 62.1% and 69.7% of patients achieved LDL‐C < 70 mg/dL, respectively.

**Conclusions:**

In this real‐world analysis, evolocumab was associated with significant reductions in LDL‐C comparable to that seen in the FOURIER clinical trial, which were durable over 1 year of treatment.

## 1 INTRODUCTION

Cardiovascular (CV) disease (CVD) remains the leading cause of death in the United States (US).^1^ The costs of CVD are rising, driven by high‐risk patients, and are expected to nearly double to $1 trillion from 2015 to 2035.^2^ Low‐density lipoprotein cholesterol (LDL‐C) is a well‐established causal risk factor for atherosclerotic CVD (ASCVD).^3^, ^4^ Accordingly, reduction of LDL‐C significantly reduces the risk of major CV events,^5^, ^6^, ^7^, ^8^, ^9^, ^10^, ^11^, ^12^ and it is now recognized that lower LDL‐C is better, with a progressive reduction in major CV events and no increase in safety events even at very low LDL‐C levels of < 7.7 mg/dL (< 0.2 mmol/L).^13^

The 2018 American College of Cardiology (ACC)/American Heart Association (AHA) Multisociety blood cholesterol guideline introduced the stratification of ''very high‐risk'' (VHR) ASCVD patients, defined as those with a history of multiple major ASCVD events (ie, recent acute coronary syndrome [ACS], history of myocardial infarction [MI], history of ischemic stroke [IS], or symptomatic peripheral arterial disease [PAD]) or a single major ASCVD event and multiple high‐risk conditions.^14^ For patients with VHR ASCVD, the 2018 ACC/AHA guideline recommends using an LDL‐C threshold of 70 mg/dL (1.8 mmol/L) to initiate the addition of nonstatins (ie, ezetimibe and proprotein convertase subtilisin/kexin type 9 [PCSK9] inhibitors) to maximally tolerated statin therapy.^14^ The European Society of Cardiology (ESC)/European Atherosclerosis Society (EAS) guidelines recommend a ≥ 50% LDL‐C reduction from baseline and a goal of < 55 mg/dL (< 1.4 mmol/L), with a lower goal of < 40 mg/dL (< 1.0 mmol/L) for patients who experience a second vascular event within 2 years while taking maximally tolerated statin therapy.^15^ The American Association of Clinical Endocrinology (AACE) and American College of Endocrinology (ACE) now recommend LDL‐C goals of < 70 mg/dL (< 1.8 mmol/L) and < 55 mg/dL (< 1.4 mmol/L) for VHR and extreme‐risk patients, respectively.^16^

However, observational studies indicate that patients infrequently achieve these guideline‐recommended LDL‐C goals in daily clinical practice,^17^, ^18^, ^19^, ^20^, ^21^ with a recent analysis of US adults with health insurance in the MarketScan^®^ database reporting that 67% of patients with VHR ASCVD had LDL‐C ≥ 70 mg/dL (≥ 1.8 mmol/L).^21^ Multiple factors are thought to underpin this unmet treatment need, with suboptimal dosing, high rates of treatment discontinuation, and low rates of lipid‐lowering therapy (LLT) adherence commonly observed.^22^, ^23^, ^24^, ^25^, ^26^, ^27^

Evolocumab is a PCSK9 inhibitor antibody approved to reduce the risk of MI, stroke, and coronary revascularization in adults with established CVD.^28^ The efficacy of evolocumab is well established from clinical trials, with significant lowering of LDL‐C and reduction of major CV events.^5^, ^9^, ^10^, ^11^, ^12^, ^13^ In the Further Cardiovascular Outcomes Research with PCSK9 Inhibition in Subjects with Elevated Risk (FOURIER) trial, the addition of evolocumab to statin therapy in patients with stable ASCVD reduced LDL‐C by a mean of 59% and significantly reduced the risk of the primary endpoints (CV death, MI, stroke, hospitalization for unstable angina, or coronary revascularization) by 15% at 48 weeks.^5^ When assessed individually, evolocumab significantly reduced the risk of MI and stroke by 27% and 21%, respectively.^5^ Secondary analyses from FOURIER report that patients with a recent MI are at an increased risk of CV events and experience greater relative and absolute risk reductions with evolocumab versus those with more remote MIs.^12^, ^29^ For example, evolocumab significantly reduced the risk of the key secondary endpoints (CV death, MI, or stroke) by 25% in patients with a recent MI (< 1 year) versus 15% in patients with a remote MI (> 1 year).^29^ Within the context of personalized medicine, these data suggest that it is reasonable to target evolocumab therapy to higher‐risk patients following a recent MI.^12^, ^29^ However, there are limited data on LDL‐C lowering with evolocumab in real‐world healthcare settings, where patient‐, physician‐, and payer‐related treatment hurdles are common.^17^, ^22^, ^23^, ^24^, ^25^, ^26^, ^27^, ^30^, ^31^, ^32^, ^33^ Historically, these hurdles have included treatment discontinuation, educational needs for providers, prior authorization requirements, costs, and formulary restrictions.^30^, ^31^, ^32^, ^33^

In the current study, we characterized the population and assessed LDL‐C lowering during 1 year of evolocumab therapy in real‐world patients with LDL‐C ≥ 70 mg/dL (≥ 1.8 mmol/L), including a patient subgroup with elevated risk hospitalized for MI within 12 months prior to starting evolocumab.

## 2 METHODS

### 2.1 Study design and patients

This was a retrospective cohort study using clinical laboratory data (Prognos, New York, NY), linked to medical and prescription claims data (IQVIA open source pharmacy claims [Dx/LRx] and adjudicated claims [PharMetrics Plus^®^] databases; IQVIA, Plymouth Meeting, PA). The study timeframe was from July 1, 2014, to November 30, 2019. Patients with a prescription claim for evolocumab between July 1, 2015, and October 31, 2019, were identified. The index date was defined as the patient's first fill for evolocumab during this period. The pre‐index (baseline) period was the 12‐month period preceding the index event, during which baseline demographics and clinical characteristics were collected. Patients were followed‐up for a minimum of 1 month up to 12 months after initiation of evolocumab or until evolocumab discontinuation (defined as a gap of > 30 or > 60 days in the overall cohort and recent MI subgroup, respectively, between the end of days' supply from a prescription to the next expected fill date).

Patients were required to be ≥ 18 years of age at the index date, have sufficient pharmacy and medical claims data during the 12 months pre‐index, have a pre‐index LDL‐C (the highest value recorded during the 6 months pre‐index) ≥ 70 mg/dL (≥ 1.8 mmol/L), and no gap in evolocumab use of > 30 days from the expected fill date before the first post‐index LDL‐C. The full list of inclusion criteria is shown in Supplementary Figure 1. Additionally, we identified a patient subgroup with an inpatient claim for MI within 12 months before the first evolocumab fill. A > 60‐day gap in evolocumab use was used as the inclusion criterion for the recent MI subgroup, in order to maximize the sample size (Supplementary Figure 1).

As the study used only existing de‐identified patient records, institutional review board approval and patient informed consent were not required. All authors had full access to the study data and take responsibility for its integrity. Investigations were in accordance with the Declaration of Helsinki.

### 2.2 Outcomes

We assessed baseline demographics (age, sex, insurance type) and clinical characteristics, including ASCVD diagnosis (MI, unstable angina, stroke [IS or transient ischemic attack], coronary revascularization, PAD, or other ASCVD [Supplementary Table 1 lists the International Classification of Diseases, Current Procedural Terminology, and Healthcare Common Procedure Coding System codes used for ASCVD diagnosis]), comorbidities, LDL‐C levels, and LLT utilization. The median of the lowest post‐index LDL‐C for each cohort was calculated from the lowest LDL‐C value recorded for each patient during the 12‐month follow‐up period or until treatment discontinuation. Percent change from baseline median LDL‐C to the lowest post‐index median LDL‐C was also calculated for each cohort. The proportions of patients meeting LDL‐C thresholds (< 40 mg/dL [< 1.0 mmol/L], < 55 mg/dL [< 1.4 mmol/L], < 70 mg/dL [< 1.8 mmol/L], and < 70 mg/dL [< 1.8 mmol/L] or a ≥ 50% reduction) were assessed, based on the lowest post‐index LDL‐C value recorded during the 12‐month follow‐up period. The proportions of patients meeting LDL‐C thresholds were also calculated in patients with baseline LDL‐C 70–189 mg/dL (1.8–4.9 mmol/L) to assess LDL‐C lowering in patients without probable familial hypercholesterolemia (eg, LDL‐C ≥ 190 mg/dL [≥ 4.9 mmol/L]).

### 2.3 Statistical analysis

The study was not designed to make formal statistical comparisons and was descriptive in nature. Mean (standard deviation [SD]) and median (interquartile range [IQR]) were calculated for continuous variables. Frequencies and percentages were calculated for categorical variables.

## 3 RESULTS

### 3.1 Baseline demographics and clinical characteristics

The study included 5897 patients with LDL‐C ≥ 70 mg/dL (≥ 1.8 mmol/L) who initiated evolocumab and 152 patients hospitalized for MI within 12 months prior to initiating evolocumab. The mean (SD) age of the overall cohort was 65 (10) years; 51.2% of patients were men, 61.9% of patients had commercial insurance, and 36.4% of patients had Medicare (Part C and D) coverage. Demographics of the recent MI subgroup were similar, but with more men than in the overall cohort (Table 1).

**TABLE 1:** Baseline demographic characteristics

Demographic characteristic	Overall evolocumab cohort (N = 5897)	Recent MI subgroup (N = 152)
Age, mean (SD), years	65 (10)	65 (10)
Age, median, years	65	63
Age ≥ 65 years, %	53.6	46.7
Sex, male, %	51.2	61.2
Payer type, %		
Commercial	61.9	63.2
Medicare (Part C and D)	36.4	36.8
Other	1.7	0.0

*Note*: A > 60‐day treatment gap was used to define discontinuation for the recent MI subgroup. MI, myocardial infarction; SD, standard deviation.

In the overall cohort, 61.9% of patients had a history of ASCVD (Table 2). Per the 2018 ACC/AHA guideline,^14^ 94.6% of patients had at least 1 high‐risk condition (Table 2). During the 12‐month baseline period, 70.7% and 78.3% of patients in the overall cohort and recent MI subgroup, respectively, were in receipt of any LLT; 60.6% and 69.1% of patients in the overall cohort and recent MI subgroup, respectively, were in receipt of any statin. High‐intensity statins were used in 26.6% and 38.2% of patients in the overall cohort and recent MI subgroup, respectively (Table 2).

**TABLE 2:** Baseline clinical characteristics

Clinical characteristic	Overall evolocumab cohort (N = 5897)	Recent MI subgroup (N = 152)
CCI, mean (SD)	2.0 (2.1)	4.1 (2.5)
LDL‐C, mean (SD), mg/dL, [mmol/L]	152 (51) [3.9 (1.3)]	140 (41) [3.6 (1.1)]
LDL‐C, median (IQR), mg/dL, [mmol/L]	146 (115–180) [3.8 (3.0–4.7)]	137 (109–165) [3.5 (2.8–4.3)]
LDL‐C category, %		
70–99 mg/dL (1.8–2.6 mmol/L)	13.7	17.1
100–129 mg/dL (2.6–3.3 mmol/L)	22.5	23.7
130–189 mg/dL (3.4–4.9 mmol/L)	43.5	49.3
≥ 190 mg/dL (≥ 4.9 mmol/L)	20.3	9.9
Probable FH^a^, %	36.1	31.6
Baseline LLT, %		
Any statin/ezetimibe	70.7	78.3
Any statin	60.6	69.1
Statin only	38.6	45.4
High‐intensity statin	15.5	28.3
Medium‐intensity statin	18.4	12.5
Low‐intensity statin	4.7	4.6
Statin plus ezetimibe	22.0	23.7
High‐intensity statin	11.1	9.9
Medium‐intensity statin	8.5	9.9
Low‐intensity statin	2.5	4.0
Ezetimibe only	10.1	9.2
ASCVD diagnosis, %		
At least 1 ASCVD diagnosis, including other ASCVD^b^	61.9	100.0
MI	20.4	100.0
UA	3.7	24.3
Stroke (IS or TIA)	7.4	10.5
Revascularization (PCI, CABG, other)	20.0	84.9
PAD	23.1	19.1
Other ASCVD^b^	85.6	93.4
ACC/AHA high‐risk conditions, %		
At least 1 ACC/AHA high‐risk condition	94.6	98.0
Age ≥ 65 years	53.6	46.7
HeFH	14.8	7.2
History of prior CABG/PCI	9.8	67.1
Diabetes mellitus	35.7	48.7
Hypertension	53.9	83.6
CKD	5.8	18.4
Current smoking	4.5	15.8
Persistently elevated LDL‐C ≥ 100 mg/dL (≥ 2.6 mmol/L) despite maximally tolerated statin and ezetimibe	66.8	64.5
History of congestive heart failure	7.0	31.6
At least 1 MACE^c^, %	8.1	90.1

^a^ Pre‐index LDL‐C ≥ 190 mg/dL (≥4.9 mmol/L) or ICD‐10 Dx code E78.01.

^b^ Any condition that was listed under ASCVD other than MI, UA, stroke (IS or TIA), revascularization, or PAD.

^c^ MACE defined as MI, UA, revascularization, or IS only. A > 60‐day treatment gap was used to define discontinuation for the recent MI subgroup. ACC, American College of Cardiology; AHA, American Heart Association; ASCVD, atherosclerotic cardiovascular disease; CABG, coronary artery bypass graft; CCI, Charlson Comorbidity Index; CKD, chronic kidney disease; FH, familial hypercholesterolemia; HeFH, heterozygous familial hypercholesterolemia; ICD, International Classification of Diseases; IQR, interquartile range; IS, ischemic stroke; LDL‐C, low‐density lipoprotein cholesterol; LLT, lipid‐lowering therapy; MACE, major adverse cardiovascular event; MI, myocardial infarction; PAD, peripheral arterial disease; PCI, percutaneous coronary intervention; SD, standard deviation; TIA, transient ischemic attack; UA, unstable angina.

### 3.2 LDL‐C reduction with evolocumab in the overall cohort

**FIGURE 1:**
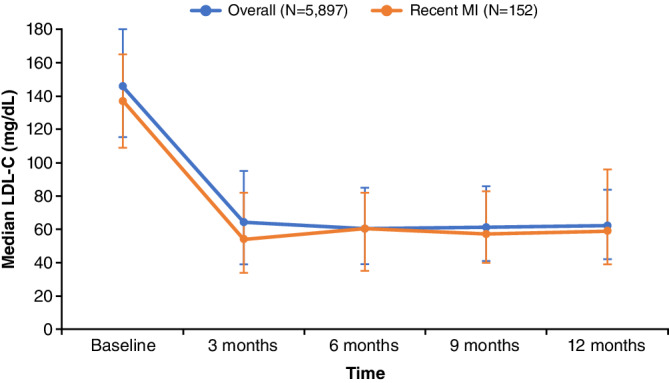
Reduction in median LDL‐C from baseline over 1 year of treatment with evolocumab in the overall cohort and recent MI subgroup. Vertical bars represent interquartile ranges. LDL‐C, low‐density lipoprotein cholesterol; MI, myocardial infarction

**FIGURE 2:**
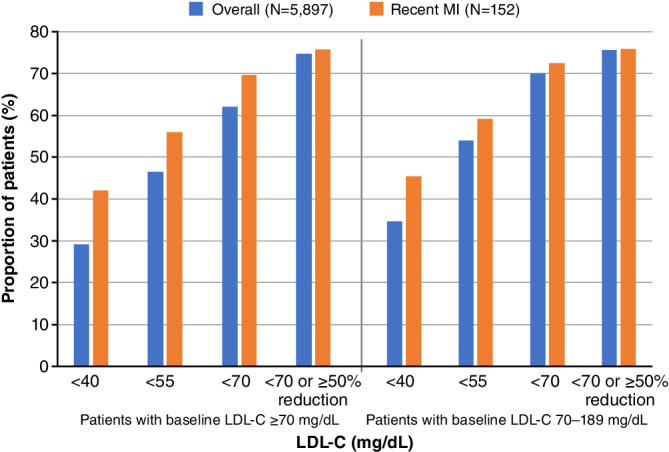
Post‐index LDL‐C thresholds after initiating evolocumab in the overall cohort and recent MI subgroup. Based on the lowest LDL‐C value measured within 12 months after evolocumab initiation. LDL‐C, low‐density lipoprotein cholesterol; MI, myocardial infarction

The baseline median (IQR) LDL‐C was 146 (115–180) mg/dL (3.8 [3.0–4.7] mmol/L) (Table 2; Figure 1). The median (IQR) follow‐up time was 213 (106–365) days. Following the initiation of evolocumab, median (IQR) LDL‐C levels decreased in the first 3 months to 64 (39–95) mg/dL (1.7 [1.0–2.5] mmol/L), and this reduction was maintained over the subsequent 9 months of therapy (Figure 1). The lowest median (IQR) LDL‐C value observed during the follow‐up period was 58 (36–84) mg/dL (1.5 [0.9–2.2] mmol/L), corresponding to a −60% change in LDL‐C from baseline to the lowest median LDL‐C recorded with evolocumab treatment. In the first 3 months of treatment, 55.4% of patients achieved LDL‐C < 70 mg/dL (< 1.8 mmol/L) and 67.7% of patients achieved LDL‐C < 70 mg/dL (< 1.8 mmol/L) or a ≥ 50% reduction in LDL‐C. Over 1 year of treatment, 62.1% of patients achieved LDL‐C < 70 mg/dL (< 1.8 mmol/L) and 74.6% of patients achieved LDL‐C < 70 mg/dL (< 1.8 mmol/L) or a ≥ 50% reduction in LDL‐C (Figure 2). Of those with baseline LDL‐C 70–189 mg/dL (1.8–4.9 mmol/L), 69.9% of patients achieved LDL‐C < 70 mg/dL (< 1.8 mmol/L) and 54.0% of patients achieved LDL‐C < 55 mg/dL (< 1.4 mmol/L); 75.6% of patients achieved LDL‐C < 70 mg/dL (< 1.8 mmol/L) or a ≥ 50% reduction in LDL‐C (Figure 2). Of those with probable familial hypercholesterolemia at baseline (eg, LDL‐C ≥ 190 mg/dL [≥ 4.9 mmol/L]), 31.3% of patients achieved LDL‐C < 70 mg/dL (< 1.8 mmol/L) and 17.3% of patients achieved LDL‐C < 55 mg/dL (< 1.4 mmol/L); 70.9% of patients achieved LDL‐C < 70 mg/dL (< 1.8 mmol/L) or a ≥ 50% reduction in LDL‐C.

### 3.3 LDL‐C reduction with evolocumab following recent MI

The median (IQR) time from MI to the initiation of evolocumab was 130 (69–234) days. A similar pattern of early and durable lowering of LDL‐C over 1 year of therapy was observed in patients with a recent MI (Figure 1). Median (IQR) LDL‐C reduced from 137 (109–165) mg/dL (3.5 [2.8–4.3] mmol/L) at baseline to the lowest median (IQR) of 48 (30–78) mg/dL (1.2 [0.8–2.0] mmol/L) during the follow‐up period, corresponding to a −65% change in LDL‐C from baseline to the lowest median LDL‐C recorded with evolocumab treatment. Over 1 year of treatment, 69.7% of patients achieved LDL‐C < 70 mg/dL (< 1.8 mmol/L) and 75.7% of patients achieved LDL‐C < 70 mg/dL (< 1.8 mmol/L) or a ≥ 50% reduction in LDL‐C. Of those with baseline LDL‐C 70–189 mg/dL (1.8–4.9 mmol/L), 72.3% of patients achieved LDL‐C < 70 mg/dL (< 1.8 mmol/L) and 59.1% of patients achieved LDL‐C < 55 mg/dL (< 1.4 mmol/L); 75.9% of patients achieved LDL‐C < 70 mg/dL (< 1.8 mmol/L) or a ≥ 50% reduction in LDL‐C (Figure 2).

## 4 DISCUSSION

This real‐world analysis describes the characteristics and LDL‐C outcomes of patients who initiated evolocumab therapy between July 1, 2015, and October 31, 2019, in US healthcare settings, including patients with a recent MI within 12 months (median 130 days) prior to starting evolocumab therapy. Overall, patient clinical characteristics were largely consistent with the recommendation that patients selected for PCSK9 inhibitor therapy are those with high levels of CV risk,^14^, ^15^, ^16^ and supports a previous real‐world study reporting that physicians were most likely to prescribe PCSK9 inhibitors to patients with elevated LDL‐C levels and additional comorbid risk factors for adverse CV events.^34^

At baseline, LDL‐C levels were markedly elevated despite the majority of patients receiving background LLT with statins and/or ezetimibe in the 12 months prior to evolocumab initiation. Of note, the 60.6% of patients treated with statins prior to evolocumab initiation in the current study is considerably higher than that reported in a recently published study where only 29.3% of patients filled a statin in the period 31–365 days prior to PCSK9 inhibitor initiation.^35^ The relatively higher proportion of patients treated with statins in the current study may be accounted for by lower levels of statin intolerance versus earlier patient cohorts who initiated PCSK9 inhibitors following their Food and Drug Administration (FDA) approval in 2015^35^; however, statin intolerance was not assessed in the current study and therefore firm conclusions should be avoided.

In the overall cohort, evolocumab therapy resulted in clinically relevant LDL‐C reductions in line with current guideline recommendations.^14^, ^15^, ^16^ Notably, the reduction from baseline to the lowest recorded median LDL‐C was 60%, and despite the high baseline LDL‐C, 62.1% of patients achieved LDL‐C < 70 mg/dL (< 1.8 mmol/L). Moreover, 70.9% of patients with probable familial hypercholesterolemia at baseline (eg, LDL‐C ≥ 190 mg/dL [≥ 4.9 mmol/L]), achieved LDL‐C < 70 mg/dL (< 1.8 mmol/L) or a ≥ 50% reduction in LDL‐C; however, the benefits of evolocumab were also consistent in patients without probable familial hypercholesterolemia at baseline. The percent reduction in LDL‐C observed in the current real‐world study is in line with that observed in previous evolocumab trials,^5^, ^10^, ^11^ including the 59% reduction in LDL‐C reported in the FOURIER trial.^5^ Moreover, the current results demonstrate the durability of the LDL‐C lowering effect of evolocumab, with the large initial reduction in median LDL‐C during the first 3 months maintained over the subsequent 9 months of therapy in real‐world clinical practice. The proportions of patients who achieved LDL‐C < 70 mg/dL (< 1.8 mmol/L) and < 55 mg/dL (< 1.4 mmol/L) in the current study are higher than in a recently reported real‐world analysis of PCSK9 inhibitor initiation between January 2016 and December 2019 in a large health maintenance organization, where 55% and 38% of patients with established CVD achieved LDL‐C < 70 mg/dL (< 1.8 mmol/L) and < 55 mg/dL (< 1.4 mmol/L), respectively.^33^

The current study provides novel and pertinent real‐world data on evolocumab therapy in patients at the highest level of CV risk. Literature suggests that substantial residual risk for MI and coronary heart disease events remains after an MI, despite intensive guideline‐directed medical management including high‐intensity statins.^36^ Thus, treatment to reduce CV risk during this period is critical, with it now recognized that patients at the highest levels of CV risk are likely to derive greater benefit from PCSK9 inhibition.^12^, ^29^, ^37^ Here, we assessed a patient subgroup with a median of 130 days between MI and evolocumab initiation. Despite a recent MI, these patients had highly elevated LDL‐C, exposing them to a high residual CV risk and confirming a pervasive unmet treatment need in this patient population. Similar to the overall cohort, evolocumab demonstrated clinically relevant LDL‐C lowering, with a 65% reduction from baseline to the lowest recorded median LDL‐C. Moreover, 69.7% of patients achieved LDL‐C < 70 mg/dL (< 1.8 mmol/L), and 75.7% of patients achieved LDL‐C < 70 mg/dL (< 1.8 mmol/L) or a ≥ 50% reduction. Notably, the proportion of patients with a recent MI who achieved LDL‐C < 70 mg/dL (< 1.8 mmol/L) with evolocumab is approximately 3 times higher than that in existing real‐world studies of LLT in patients with elevated CV risk (ie, recent ACS).^18^, ^19^, ^20^ Therefore, the current results indicate that initiating evolocumab therapy soon after an MI to achieve early, intensive, and durable LDL‐C lowering may address the unmet treatment need in this patient population. Indeed, a secondary analysis from the FOURIER trial recently reported that 91.7% of patients with a recent MI (< 1 year) achieved LDL‐C < 70 mg/dL (< 1.8 mmol/L) after 4 weeks of evolocumab treatment.^29^

The Evolocumab for Early Reduction of LDL‐Cholesterol Levels in Patients With Acute Coronary Syndromes (EVOPACS) trial was the first to assess PCSK9 inhibitor antibody treatment during the acute phase of ACS.^38^ After 8 weeks of evolocumab treatment, 95.7% of patients achieved LDL‐C < 70 mg/dL (< 1.8 mmol/L) and the mean reduction in LDL‐C from baseline was 77.1%.^38^ A higher proportion of patients achieved LDL‐C < 70 mg/dL (< 1.8 mmol/L) with evolocumab in the EVOPACS trial than in the current study; however, there are salient differences in study design that may account for the difference. Specifically, EVOPACS was a randomized, placebo‐controlled trial that combined evolocumab with a high‐intensity statin in a predominately statin‐naïve population.^38^ Thus, larger reductions in LDL‐C may be expected than in the current study, where most patients were in receipt of LLT at baseline but not necessarily during the evolocumab treatment period, as concomitant LLT was not a study requirement.

### 4.1 Limitations

The current study has a number of limitations. Firstly, medical history among patients in the LRx database may have been under‐captured due to the open nature of the data and no requirement for continuous coverage at the patient level. Secondly, it was subject to the established limitations of real‐world research using claims databases, including limited generalizability to patients without health insurance. Thirdly, as evolocumab therapy was ascertained from documented prescriptions, it was not clear if the patient used the product on the expected schedule. Fourthly, it has been reported that the most common physician‐reported reason for initiating PCSK9 inhibitor treatment in real‐world clinical practice is a lack of statin efficacy^39^; however, in the current study we were unable to assess physician and patient factors (eg, statin intolerance) influencing treatment choice. Fifth, the recent MI subgroup was relatively small. Finally, the current study ended shortly after the 2018 ACC/AHA blood cholesterol guideline was published^14^; therefore, putative changes in treatment patterns arising from the adoption of the guideline updates were unable to be fully observed in this study.

## 5 CONCLUSIONS

In conclusion, many patients initiating evolocumab therapy in US clinical practice had highly elevated LDL‐C, and high‐risk comorbidities and/or ASCVD were common. Consistent with previous clinical studies,^5^, ^10^, ^11^ in the real‐world, evolocumab demonstrated clinically relevant LDL‐C reductions that were maintained over 1 year of treatment for all initiators and among the highest‐risk patients with a prior MI.

## CONFLICT OF INTEREST

Nihar R. Desai reports research grants from Medtronic, Johnson & Johnson; consulting fees from Amgen Inc., Relypsa, OPKO, scPharmaceuticals, and Cytokinetics. Rolin L. Wade, Xin Wang, Chi‐Chang Chen are employees of IQVIA, which was hired by Amgen to conduct this study. Pin Xiang was an employee of Amgen Inc. when the study was conducted and is an employee of Boehringer Ingelheim. Sasikiran Nunna was an employee of IQVIA when the study was conducted and is an employee of Bristol Myers Squibb. Lionel Pinto, Jason Exter, Katherine E. Mues, Mohdhar Habib are employees of Amgen Inc. and own Amgen stock.

## Supporting information


Appendix S1: Supporting information

## Data Availability

Qualified researchers may request data from Amgen clinical studies. Complete details are available at the following: http://www.amgen.com/datasharing.
