# Supplementary material for: Low‐density lipoprotein cholesterol lowering in real‐world patients treated with evolocumab
Source: Clin Cardiol. 2021 Mar 24;44(5):715–22. doi: 10.1002/clc.23600 (PMC8119857; doi:10.1002/clc.23600)
Supplement: Supplementary file 1 — Appendix S1: Supporting information [file CLC-44-715-s001.docx]

**SUPPLEMENTAL MATERIAL**

Supplementary Table 1. ASCVD Diagnosis Codes

| **ASCVD Diagnosis** | **Codes** |
| --- | --- |
| Myocardial infarction | **ICD-10-CM:** I21.01, I21.02, I21.09, I21.11, I21.21, I21.29, I21.3, I21.4, I22.0, I22.1, I22.2, I22.8, I22.9, I25.2, I23.0, I23.1, I23.2, I23.3, I23.4, I23.5, I23.6, I23.7, I23.8  **ICD-9:** 410.00, 410.01, 410.02, 410.10, 410.11, 410.12, 410.20, 410.21, 410.22, 410.30, 410.31, 410.32, 410.40, 410.41, 410.42, 410.50, 410.51, 410.52, 410.60, 410.61, 410.62, 410.70, 410.71, 410.72, 410.80, 410.81, 410.82, 410.90, 410.91, 410.92, 412, 429.79, 411.0 |
| Unstable angina | **ICD-10-CM:** I20.0, I20.1, I20.8, I20.9, I23.7, I24.0, I24.8, I25.110, I25.111, I25.118, I25.119, I25.700, I25.701, I25.708, I25.709, I25.710, I25.711, I25.718, I25.719, I25.720, I25.721, I25.728, I25.729, I25.730, I25.731, I25.738, I25.739, I25.750, I25.751, I25.758, I25.759, I25.760, I25.761, I25.768, I25.769, I25.790, I25.791, I25.798, I25.799  **ICD-9:** 411.1, 411.81, 411.89, 413.9, 413.0 |
| Ischemic stroke | **ICD-10-CM:** Z86.73, I63.00, I63.011, I63.012, I63.013, I63.019, I63.02, I63.031, I63.032, I63.033, I63.039, I63.09, I63.20, I63.211, I63.212, I63.213, I63.219, I63.22, I63.231, I63.232, I63.233, I63.239, I63.29, I63.30, I63.311, I63.312, I63.313, I63.319, I63.321, I63.322, I63.323, I63.329, I63.331, I63.332, I63.333, I63.339, I63.341, I63.342, I63.343, I63.349, I63.39, I63.50, I63.511, I63.512, I63.513, I63.519, I63.521, I63.522, I63.523, I63.529, I62.531, I63.532, I63.533, I63.539, I63.541, I63.542, I63.543, I63.549, I63.59, I63.6, I63.8, I63.9  **ICD-9-CM:** 434.01, 434.91, 433.01, 433.11, 433.21, 433.31, 433.81, 433.91, V12.54, 436 |
| Transient ischemic attack | **ICD-10-CM:** G45.0, G45.1, G45.2, G45.3, G45.8, G45.9, G46.0, G46.1, H34.00, H34.01, H34.02, H34.03, I67.841, I67.848  **ICD-9-CM:** 362.34, 435.0, 435.1, 435.2, 435.3, 435.8, 435.9 |
| Coronary revascularization (percutaneous coronary intervention) | **ICD-10-CM:** Z98.61, Z95.5, T82.855A, T82.855D, T82.855S  **ICD-10-PCS:** 0270346, 027034Z, 0270356, 027035Z, 0270366, 027036Z, 0270376, 027037Z, 02703D6, 02703DZ, 02703E6, 02703EZ, 02703F6, 02703FZ, 02703G6, 02703GZ, 02703T6, 02703TZ, 02703Z6, 02703ZZ, 0270446, 027044Z, 0270456, 027045Z, 0270466, 027046Z, 0270476, 027047Z, 02704D6, 02704DZ, 02704E6, 02704EZ, 2704F6, 02704FZ, 02704G6, 02704GZ, 02704T6, 02704TZ, 02704Z6, 02704ZZ, 0271346, 027134Z, 0271356, 027135Z, 0271366, 027136Z,0271376, 027137Z, 02713D6, 02713DZ, 02713E6, 02713EZ, 02713F6, 02713FZ, 02713G6, 02713GZ, 02713T6, 02713TZ, 02713Z6, 02713ZZ, 0271446, 027144Z, 0271456, 027145Z, 0271466, 027146Z, 0271476, 027147Z, 02714D6, 02714DZ, 02714E6, 02714EZ, 02714F6, 02714FZ, 02714G6, 02714GZ, 02714T6, 02714TZ, 02714Z6, 02714ZZ, 0272346, 027234Z, 0272356, 027235Z, 0272366, 027236Z, 0272376, 027237Z, 02723D6, 02723DZ, 02723E6, 02723EZ, 02723F6, 02723FZ, 02723G6, 02723GZ, 02723T6, 02723TZ, 02723Z6, 02723ZZ, 0272446, 027244Z, 0272456, 027245Z, 0272466, 027246Z, 0272476, 027247Z, 02724D6, 02724DZ, 02724E6, 02724EZ, 02724F6, 02724FZ, 02724G6,02724GZ, 02724T6, 02724TZ, 02724Z6, 02724ZZ, 0273346, 027334Z, 0273356, 027335Z, 0273366, 027336Z, 0273376, 027337Z, 02733D6, 02733DZ, 02733E6, 02733EZ, 02733F6, 02733FZ, 02733G6, 02733GZ, 02733T6, 02733TZ, 02733Z6, 02733ZZ, 0273446, 027344Z, 0273456, 027345Z, 0273466, 027346Z, 0273476, 027347Z, 02734D6, 02734DZ, 02734E6, 02734EZ, 02734F6, 02734FZ, 02734G6, 02734GZ, 02734T6, 02734TZ, 02734Z6, 02734ZZ, 02C03ZZ, 02C04ZZ, 02C13ZZ, 02C14ZZ, 02C23ZZ, 02C24ZZ, 02C33ZZ, 02C34ZZ, X2C0361, X2C1361, X2C2361, X2C3361  **CPT/****HCPCS:** 92973, C9600, C9601, 92920, 92921, 92928, 92929, 92924, 92925, 92933, 92934, 92937, 92938, 92941, 92943, 92944, C9602, C9603, C9604, C9605, C9606, C9607, C9608, 92975, 92977  **ICD-9:** V45.82, 00.66, 1755, 36.06, 36.07, 00.40, 00.41, 00.42, 00.43, 00.44, 00.45, 00.46, 00.47, 00.48 |
| Coronary revascularization (coronary artery bypass grafting) | **ICD-10-CM:** Z95.1  **ICD-10-PCS:** 210083, 0210088, 0210089, 021008C, 021008F, 021008W, 0210093, 0210098, 0210099, 021009C, 021009F, 021009W, 02100A3, 02100A8, 02100A9, 02100AC, 02100AF, 02100AW, 02100J3, 02100J8, 02100J9, 02100JC, 02100JF, 02100JW, 02100K3, 02100K8, 02100K9, 02100KC, 02100KF, 02100KW, 02100Z3, 02100Z8, 02100Z9, 02100ZC, 02100ZF, 0210344, 02103D4, 0210444, 0210483, 0210488, 0210489, 021048C, 021048F, 021048W, 0210493, 0210498, 0210499, 021049C, 021049F, 021049W, 02104A3, 02104A8, 02104A9, 02104AC, 02104AF, 02104AW, 02104D4, 02104J3,02104J8, 02104J9, 02104JC, 02104JF, 02104JW, 02104K3, 02104K8, 02104K9, 02104KC, 02104KF, 02104KW, 02104Z3, 02104Z8, 02104Z9, 02104ZC, 02104ZF, 0211083, 0211088, 0211089, 021108C, 021108F, 021108W, 0211093, 0211098, 0211099, 021109C, 021109F, 021109W, 02110A3, 02110A8, 02110A9, 02110AC, 02110AF, 02110AW, 02110J3, 02110J8, 02110J9, 02110JC, 02110JF, 02110JW, 02110K3,02110K8, 02110K9, 02110KC, 02110KF, 02110KW, 02110Z3, 02110Z8, 02110Z9, 02110ZC, 02110ZF, 0211344, 02113D4, 0211444, 0211483, 0211488, 0211489, 021148C, 021148F, 021148W, 0211493, 0211498, 0211499, 021149C, 021149F, 021149W, 02114A3, 02114A8, 02114A9, 02114AC, 02114AF, 02114AW, 02114D4, 02114J3, 02114J8, 02114J9, 02114JC, 02114JF, 02114JW, 02114K3, 02114K8, 02114K9, 02114KC, 02114KF, 02114KW, 02114Z3, 02114Z8, 02114Z9, 02114ZC, 02114ZF, 0212083, 0212088, 0212089, 021208C, 021208F, 021208W, 0212093, 0212098, 0212099, 021209C, 021209F, 021209W, 02120A3, 02120A8, 02120A9, 02120AC, 02120AF, 02120AW, 02120J3, 02120J8, 02120J9, 02120JC, 02120JF, 02120JW, 02120K3, 02120K8, 02120K9, 02120KC, 02120KF, 02120KW, 02120Z3, 02120Z8, 02120Z9, 02120ZC, 02120ZF, 0212344, 02123D4, 0212444, 0212483, 0212488, 0212489, 021248C, 021248F, 021248W, 0212493, 0212498, 0212499, 021249C, 021249F, 021249W, 02124A3, 02124A8, 02124A9, 02124AC, 02124AF, 02124AW, 02124D4, 02124J3, 02124J8, 02124J9, 02124JC, 02124JF, 02124JW, 02124K3, 02124K8, 02124K9, 02124KC, 02124KF, 02124KW, 02124Z3, 02124Z8, 02124Z9, 02124ZC, 02124ZF, 0213083, 0213088, 0213089, 021308C, 021308F, 021308W, 0213093, 0213098, 0213099, 021309C, 021309F, 021309W, 02130A3, 02130A8, 02130A9, 02130AC, 02130AF, 02130AW, 02130J3, 02130J8, 02130J9, 02130JC, 02130JF, 02130JW, 02130K3, 02130K8, 02130K9, 02130KC, 02130KF, 02130KW, 02130Z3, 02130Z8, 02130Z9, 02130ZC, 02130ZF, 0213344, 02133D4, 0213444, 0213483, 0213488, 0213489, 021348C, 021348F, 021348W, 0213493, 0213498, 0213499, 021349C, 021349F, 021349W, 02134A3, 02134A8, 02134A9, 02134AC, 02134AF, 02134AW, 02134D4, 02134J3, 02134J8, 02134J9, 02134JC, 02134JF, 02134JW, 02134K3, 02134K8, 02134K9, 02134KC, 02134KF, 02134KW, 02134Z3, 02134Z8, 02134Z9, 02134ZC, 02134ZF  **CPT/HCPCS:** 33510, 33511, 33512, 33513, 33514, 33516, 33517, 33518, 33519, 33521, 33522, 33523, 33533, 33534, 33535, 33536, 33508, S2205, S2206, S2207, S2208, S2209  **ICD-9-CM:** V45.81, 36.1, 36.10, 36.11, 36.12, 36.13, 36.14, 36.15, 36.16, 36.17, 36.19, 36.2, 36.3, 36.31, 36.32, 36.33, 36.34, 36.39 |
| Coronary revascularization (other) | **ICD-10-PCS:** 0270046, 027004Z, 02700D6, 02700DZ, 02700T6, 02700TZ, 02700Z6, 02700ZZ, 0271046, 027104Z, 02710D6, 02710DZ, 02710T6, 02710TZ, 02710Z6, 02710ZZ, 0272046, 027204Z, 02720D6, 02720DZ, 02720T6, 02720TZ, 02720Z6, 02720ZZ, 0273046, 027304Z, 02730D6, 02730DZ, 02730T6, 02730TZ, 02730Z6, 02730ZZ, 02C00ZZ, 02C10ZZ, 02C20ZZ, 02C30ZZ  **ICD-9-CM:** 36.03, 36.04, 36.09  **CPT:** 33140, 33141, 33572 |
| Peripheral arterial disease | **ICD-10-CM:** Z95.820, Z98.62, Z95.828, I70.1, I70.211, I70.212, I70.213, I70.218, I70.219, I70.221, I70.222, I70.223, I70.228, I70.229, I70.231, I70.232, I70.233, I70.234, I70.235, I70.238, I70.239, I70.241, I70.242, I70.243, I70.244, I70.245, I70.248, I70.249, I70.25, I70.261, I70.262, I70.263, I70.268, I70.269, I70.291, I70.292, I70.293, I70.298, I70.299, I70.401, I70.402, I70.403, I70.408, I70.409, I70.411, I70.412, I70.413, I70.418, I70.419, I70.421, I70.422, I70.423, I70.428, I70.429, I70.431, I70.432, I70.433, I70.434, I70.435, I70.438, I70.439, I70.441, I70.442, I70.443, I70.444, I70.445, I70.448, I70.449, I70.45, I70.461, I70.462, I70.463, I70.468, I70.469, I70.491, I70.492, I70.493, I70.498, I70.499, I70.501, I70.502, I70.503, I70.508, I70.509, I70.511, I70.512, I70.513, I70.518, I70.519, I70.521, I70.522, I70.523, I70.528, I70.529, I70.531, I70.532, I70.533, I70.534, I70.535, I70.538, I70.539, I70.541, I70.542, I70.543, I70.544, I70.545, I70.548, I70.549, I70.55, I70.561, I70.562, I70.563, I70.568, I70.569, I70.591, I70.592, I70.593, I70.598, I70.599, I70.92, I70.8, I70.90, I70.91, I75.011, I75.012, I75.013, I75.019, I75.021, I75.022, I75.023, I75.029, I75.81, I75.89, I70.0, I70.201, I70.202, I70.203, I70.208, I70.209, I70.301, I70.302, I70.303, I70.308, I70.309, I70.311, I70.312, I70.313, I70.318, I70.319, I70.321, I70.322, I70.323, I70.328, I70.329, I70.331, I70.332, I70.333, I70.334, I70.335, I70.338, I70.339, I70.341, I70.342, I70.343, I70.344, I70.345, I70.348, I70.349, I70.35, I70.361, I70.362, I70.363, I70.368, I70.369, I70.391, I70.392, I70.393, I70.398, I70.399, I70.601, I70.602, I70.603, I70.608, I70.609, I70.611, I70.612, I70.613, I70.618, I70.619, I70.621, I70.622, I70.623, I70.628, I70.629, I70.631, I70.632, I70.633, I70.634, I70.635, I70.638, I70.639, I70.641, I70.642, I70.643, I70.644, I70.645, I70.648, I70.649, I70.65, I70.661, I70.662, I70.663, I70.668, I70.669, I70.691, I70.692, I70.693, I70.698, I70.699, I70.701, I70.702, I70.703, I70.708, I70.709, I70.711, I70.712, I70.713, I70.718, I70.719, I70.721, I70.722, I70.723, I70.728, I70.729, I70.731, I70.732, I70.733, I70.734, I70.735, I70.738, I70.739, I70.741, I70.742, I70.743, I70.744, I70.745, I70.748, I70.749, I70.75, I70.761, I70.762, I70.763, I70.768, I70.769, I70.791, I70.792, I70.793, I70.798, I70.799, I73.9, I73.1, I73.89, I71.5, I71.8, I71.3, I71.1  **ICD-9-CM:** 440.0x, 440.1, 440.2x, 440.21, 440.22, 440.23, 440.24, 440.29, 440.3x, 440.30, 440.31, 440.32, 440.4x, 440.8x, 440.9x, 445.01, 445.02, 445.81, 445.89, 362.30, 440.0, 440.20, 440.30, 443.9, 441, 441.0, 441.00, 441.01, 441.02, 441.03, 441.1, 441.2, 441.3, 441.4, 441.5, 441.6, 441.7, 441.9, 442, 442.0, 442.1, 442.2, 442.3, 442.8, 442.81, 442.82, 442.83, 442.84, 442.89, 442.9, 443, 443.1, 443.2, 443.21, 443.22, 443.23, 443.24, 443.29, 443.8, 443.81, 443.89, 443.9x, 445.xx, 445.0, 445.01, 445.02, 445.8, 445.81, 445.89 |
| Other ASCVD | **ICD-10-CM:** I25.6, I25.10, I25.5, I25.810, I25.811, I25.812, I25.82, I25.83, I25.89, I25.84, I25.9  **ICD-9:** 414, 414.01, 414.02, 414.03, 414.04, 414.05, 414.06, 414.07, 414.1, 414.12, 414.19, 414.2, 414.3, 414.4, 414.8, 414.9, 429.79 |

ASCVD, atherosclerotic cardiovascular disease; CM, clinical modification; CPT, Current Procedural Terminology; HCPCS, Healthcare Common Procedure Coding System; ICD, International Classification of Diseases; PCS, Procedure Coding System.

Supplementary Figure 1. Study design


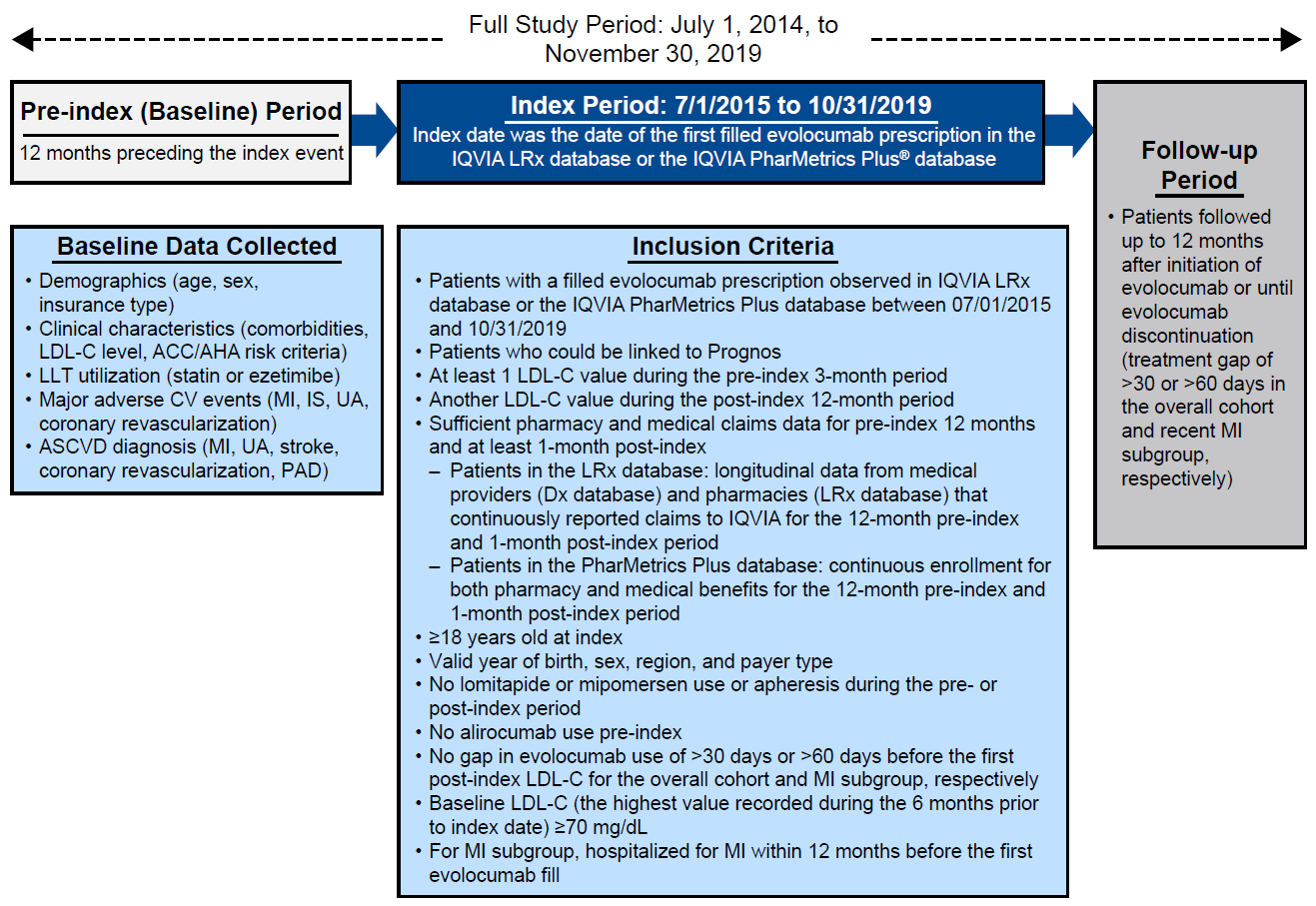


ACC, American College of Cardiology; AHA, American Heart Association; ASCVD, atherosclerotic cardiovascular disease; CV, cardiovascular; IS, ischemic stroke; LDL-C, low-density lipoprotein cholesterol; LLT, lipid-lowering therapy; MI, myocardial infarction; PAD, peripheral arterial disease; UA, unstable angina.
